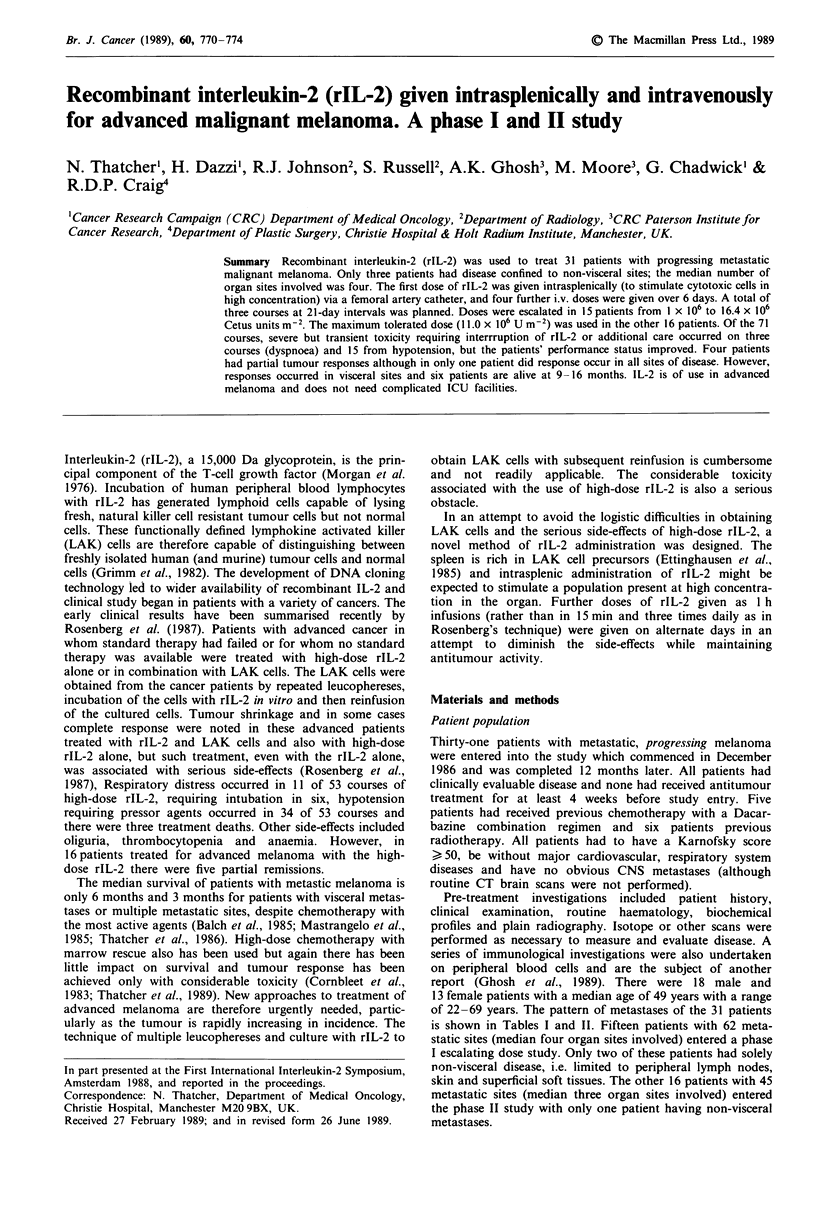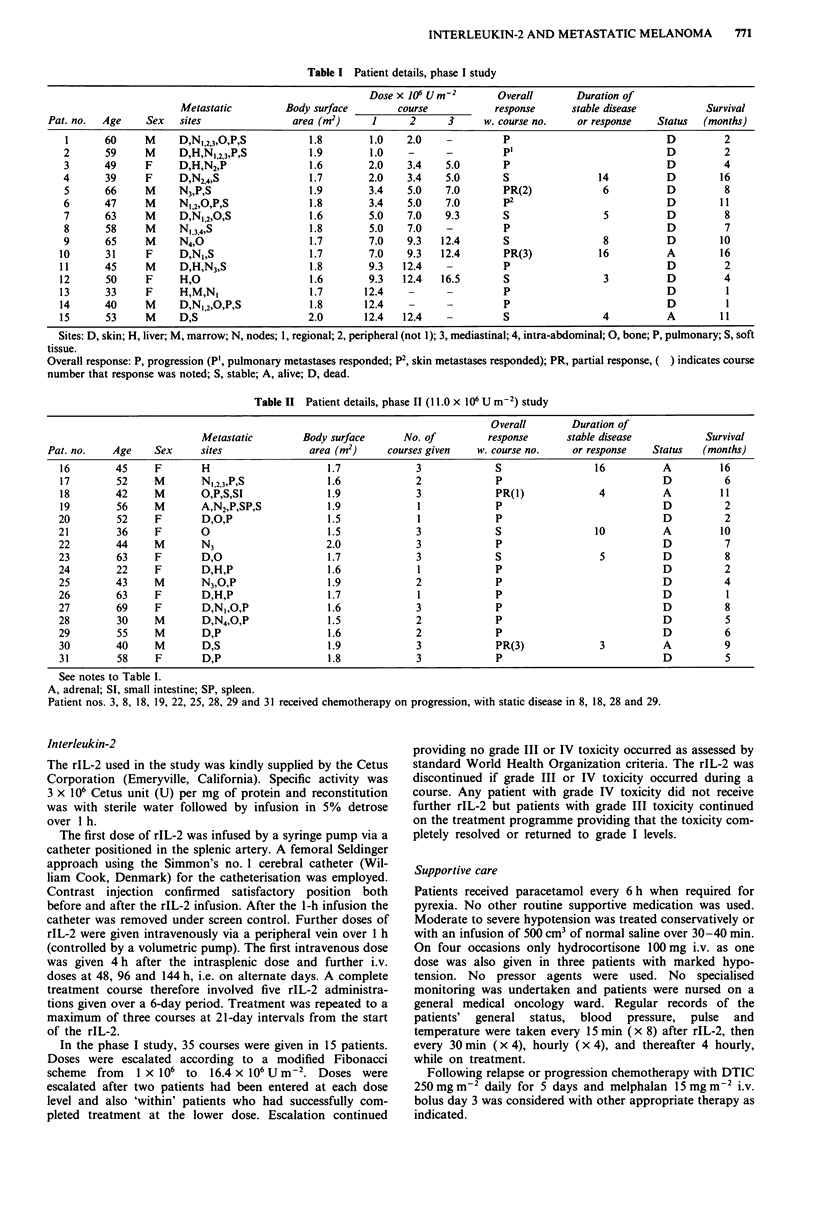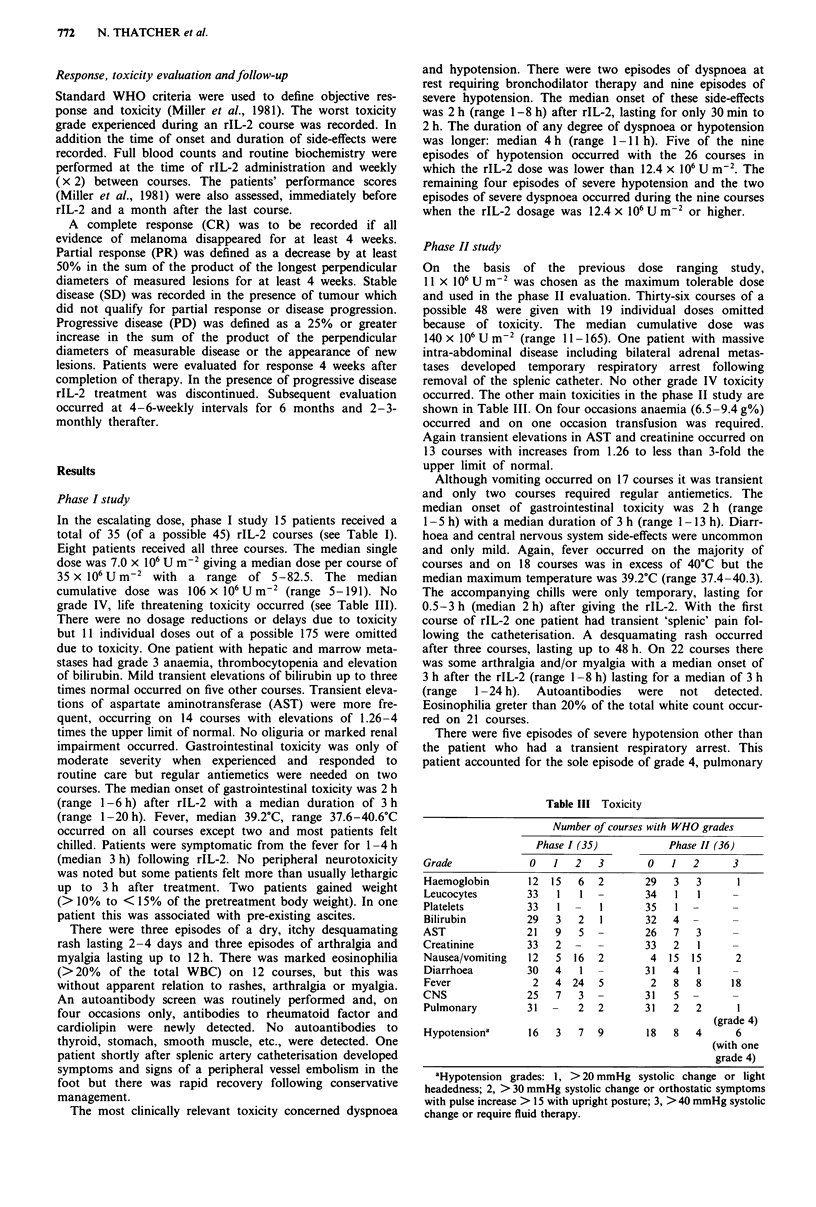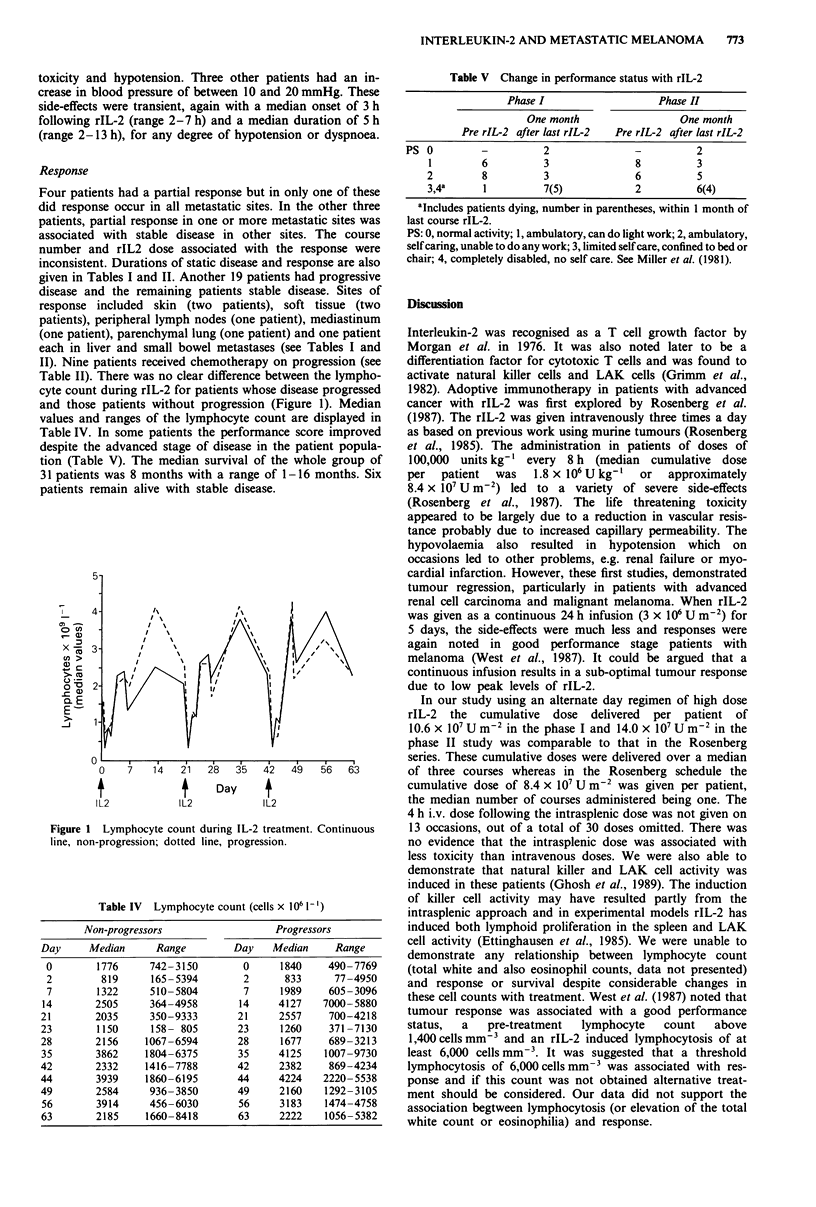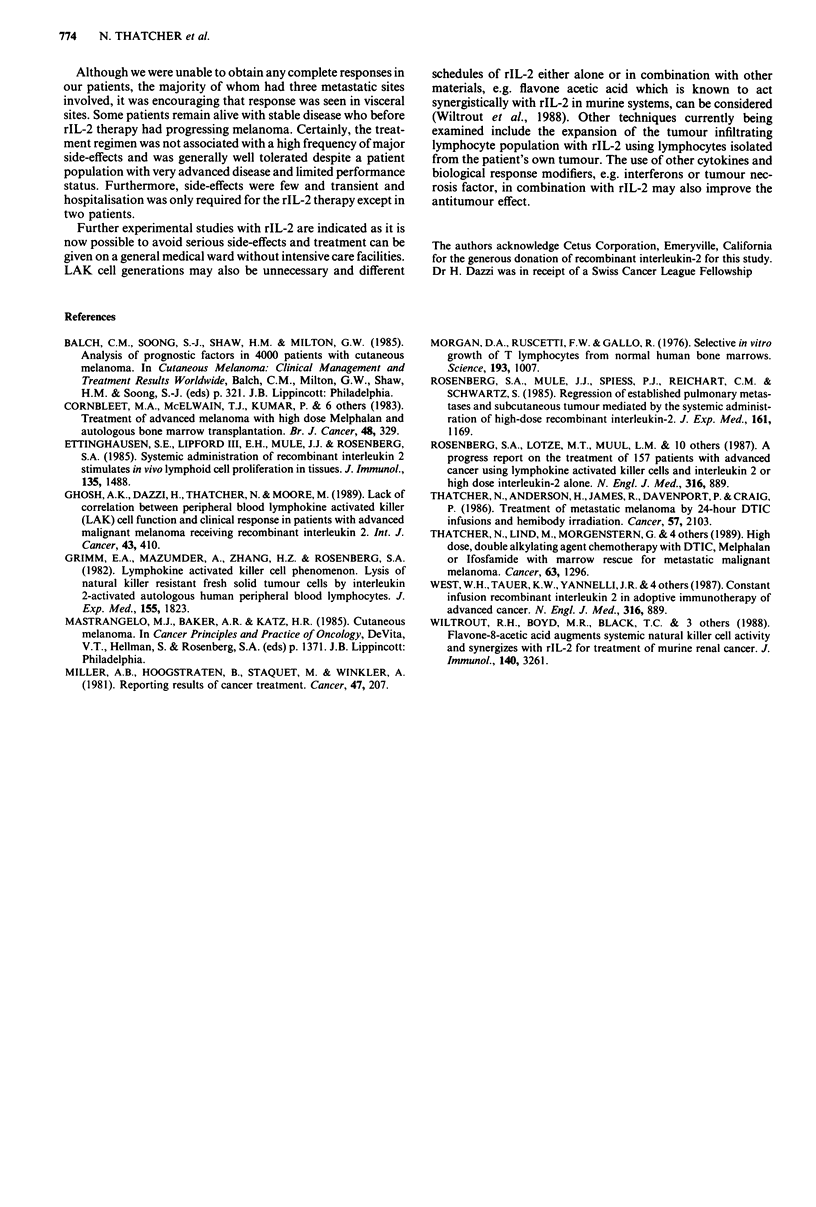# Recombinant interleukin-2 (rIL-2) given intrasplenically and intravenously for advanced malignant melanoma. A phase I and II study.

**DOI:** 10.1038/bjc.1989.357

**Published:** 1989-11

**Authors:** N. Thatcher, H. Dazzi, R. J. Johnson, S. Russell, A. K. Ghosh, M. Moore, G. Chadwick, R. D. Craig

**Affiliations:** Department of Medical Oncology, Christie Hospital & Holt Radium Institute, Manchester, UK.

## Abstract

Recombinant interleukin-2 (rIL-2) was used to treat 31 patients with progressing metastatic malignant melanoma. Only three patients had disease confined to non-visceral sites; the median number of organ sites involved was four. The first dose of rIL-2 was given intrasplenically (to stimulate cytotoxic cells in high concentration) via a femoral artery catheter, and four further i.v. doses were given over 6 days. A total of three courses at 21-day intervals was planned. Doses were escalated in 15 patients from 1 x 10(6) to 16.4 x 10(4) Cetus units m-2. The maximum tolerated dose (11.0 x 10(6) U m-2) was used in the other 16 patients. Of the 71 courses, severe but transient toxicity requiring interruption of rIL-2 or additional care occurred on three courses (dyspnoea) and 15 from hypotension, but the patients' performance status improved. Four patients had partial tumour responses although in only one patient did response occur in all sites of disease. However, responses occurred in visceral sites and six patients are alive at 9-16 months. IL-2 is of use in advanced melanoma and does not need complicated ICU facilities.


*Br. J. Cancer* (1989), **60**, 770–774 © The Macmillan Press Ltd., 1989

### Recombinant interleukin-2 (rIL-2) given intrasplenically and intravenously for advanced malignant melanoma. A phase I and II study

N. Thatcher[1], H. Dazzi[1], R.J. Johnson[2], S. Russell[2], A.K. Ghosh[3], M. Moore[3], G. Chadwick[1] & R.D.P. Craig[4]

[1]*Cancer Research Campaign (CRC) Department of Medical Oncology,* [2]*Department of Radiology,* [3]*CRC Paterson Institute for Cancer Research,* [4]*Department of Plastic Surgery, Christie Hospital & Holt Radium Institute, Manchester, UK.*

**Summary** Recombinant interleukin-2 (rIL-2) was used to treat 31 patients with progressing metastatic malignant melanoma. Only three patients had disease confined to non-visceral sites; the median number of organ sites involved was four. The first dose of rIL-2 was given intrasplenically (to stimulate cytotoxic cells in high concentration) via a femoral artery catheter, and four further i.v. doses were given over 6 days. A total of three courses at 21-day intervals was planned. Doses were escalated in 15 patients from $1 \times 10^6$ to $16.4 \times 10^6$ Cetus units $m^{-2}$. The maximum tolerated dose ($11.0 \times 10^6$ U $m^{-2}$) was used in the other 16 patients. Of the 71 courses, severe but transient toxicity requiring interrruption of rIL-2 or additional care occurred on three courses (dyspnoea) and 15 from hypotension, but the patients' performance status improved. Four patients had partial tumour responses although in only one patient did response occur in all sites of disease. However, responses occurred in visceral sites and six patients are alive at 9–16 months. IL-2 is of use in advanced melanoma and does not need complicated ICU facilities.

Interleukin-2 (rIL-2), a 15,000 Da glycoprotein, is the principal component of the T-cell growth factor (Morgan *et al.* 1976). Incubation of human peripheral blood lymphocytes with rIL-2 has generated lymphoid cells capable of lysing fresh, natural killer cell resistant tumour cells but not normal cells. These functionally defined lymphokine activated killer (LAK) cells are therefore capable of distinguishing between freshly isolated human (and murine) tumour cells and normal cells (Grimm *et al.*, 1982). The development of DNA cloning technology led to wider availability of recombinant IL-2 and clinical study began in patients with a variety of cancers. The early clinical results have been summarised recently by Rosenberg *et al.* (1987). Patients with advanced cancer in whom standard therapy had failed or for whom no standard therapy was available were treated with high-dose rIL-2 alone or in combination with LAK cells. The LAK cells were obtained from the cancer patients by repeated leucophereses, incubation of the cells with rIL-2 *in vitro* and then reinfusion of the cultured cells. Tumour shrinkage and in some cases complete response were noted in these advanced patients treated with rIL-2 and LAK cells and also with high-dose rIL-2 alone, but such treatment, even with the rIL-2 alone, was associated with serious side-effects (Rosenberg *et al.*, 1987), Respiratory distress occurred in 11 of 53 courses of high-dose rIL-2, requiring intubation in six, hypotension requiring pressor agents occurred in 34 of 53 courses and there were three treatment deaths. Other side-effects included oliguria, thrombocytopenia and anaemia. However, in 16 patients treated for advanced melanoma with the high-dose rIL-2 there were five partial remissions.

The median survival of patients with metastic melanoma is only 6 months and 3 months for patients with visceral metastases or multiple metastatic sites, despite chemotherapy with the most active agents (Balch *et al.*, 1985; Mastrangelo *et al.*, 1985; Thatcher *et al.*, 1986). High-dose chemotherapy with marrow rescue also has been used but again there has been little impact on survival and tumour response has been achieved only with considerable toxicity (Cornbleet *et al.*, 1983; Thatcher *et al.*, 1989). New approaches to treatment of advanced melanoma are therefore urgently needed, particularly as the tumour is rapidly increasing in incidence. The technique of multiple leucophereses and culture with rIL-2 to obtain LAK cells with subsequent reinfusion is cumbersome and not readily applicable. The considerable toxicity associated with the use of high-dose rIL-2 is also a serious obstacle.

In an attempt to avoid the logistic difficulties in obtaining LAK cells and the serious side-effects of high-dose rIL-2, a novel method of rIL-2 administration was designed. The spleen is rich in LAK cell precursors (Ettinghausen *et al.*, 1985) and intrasplenic administration of rIL-2 might be expected to stimulate a population present at high concentration in the organ. Further doses of rIL-2 given as 1 h infusions (rather than in 15 min and three times daily as in Rosenberg's technique) were given on alternate days in an attempt to diminish the side-effects while maintaining antitumour activity.

### Materials and methods

### *Patient population*

Thirty-one patients with metastatic, *progressing* melanoma were entered into the study which commenced in December 1986 and was completed 12 months later. All patients had clinically evaluable disease and none had received antitumour treatment for at least 4 weeks before study entry. Five patients had received previous chemotherapy with a Dacarbazine combination regimen and six patients previous radiotherapy. All patients had to have a Karnofsky score $\geqslant 50$, be without major cardiovascular, respiratory system diseases and have no obvious CNS metastases (although routine CT brain scans were not performed).

Pre-treatment investigations included patient history, clinical examination, routine haematology, biochemical profiles and plain radiography. Isotope or other scans were performed as necessary to measure and evaluate disease. A series of immunological investigations were also undertaken on peripheral blood cells and are the subject of another report (Ghosh *et al.*, 1989). There were 18 male and 13 female patients with a median age of 49 years with a range of 22–69 years. The pattern of metastases of the 31 patients is shown in Tables I and II. Fifteen patients with 62 metastatic sites (median four organ sites involved) entered a phase I escalating dose study. Only two of these patients had solely non-visceral disease, i.e. limited to peripheral lymph nodes, skin and superficial soft tissues. The other 16 patients with 45 metastatic sites (median three organ sites involved) entered the phase II study with only one patient having non-visceral metastases.

In part presented at the First International Interleukin-2 Symposium, Amsterdam 1988, and reported in the proceedings.
Correspondence: N. Thatcher, Department of Medical Oncology, Christie Hospital, Manchester M20 9BX, UK.
Received 27 February 1989; and in revised form 26 June 1989.

**Table I** Patient details, phase I study

| Pat. no. | Age | Sex | Metastatic sites | Body surface area ($m^2$) | Dose × $10^6$ U $m^{-2}$ course 1 | 2 | 3 | Overall response w. course no. | Duration of stable disease or response | Status | Survival (months) |
|---|---|---|---|---|---|---|---|---|---|---|---|
| 1 | 60 | M | D,$N_{1,2,3}$,O,P,S | 1.8 | 1.0 | 2.0 | – | P | | D | 2 |
| 2 | 59 | M | D,H,$N_{1,2,3}$,P,S | 1.9 | 1.0 | – | – | $P^1$ | | D | 2 |
| 3 | 49 | F | D,H,$N_2$,P | 1.6 | 2.0 | 3.4 | 5.0 | P | | D | 4 |
| 4 | 39 | F | D,$N_{2,4}$,S | 1.7 | 2.0 | 3.4 | 5.0 | S | 14 | D | 16 |
| 5 | 66 | M | $N_3$,P,S | 1.9 | 3.4 | 5.0 | 7.0 | PR(2) | 6 | D | 8 |
| 6 | 47 | M | $N_{1,2}$,O,P,S | 1.8 | 3.4 | 5.0 | 7.0 | $P^2$ | | D | 11 |
| 7 | 63 | M | D,$N_{1,2}$,O,S | 1.6 | 5.0 | 7.0 | 9.3 | S | 5 | D | 8 |
| 8 | 58 | M | $N_{1,3,4}$,S | 1.8 | 5.0 | 7.0 | – | P | | D | 7 |
| 9 | 65 | M | $N_4$,O | 1.7 | 7.0 | 9.3 | 12.4 | S | 8 | D | 10 |
| 10 | 31 | F | D,$N_1$,S | 1.7 | 7.0 | 9.3 | 12.4 | PR(3) | 16 | A | 16 |
| 11 | 45 | M | D,H,$N_3$,S | 1.8 | 9.3 | 12.4 | – | P | | D | 2 |
| 12 | 50 | F | H,O | 1.6 | 9.3 | 12.4 | 16.5 | S | 3 | D | 4 |
| 13 | 33 | F | H,M,$N_1$ | 1.7 | 12.4 | – | – | P | | D | 1 |
| 14 | 40 | M | D,$N_{1,2}$,O,P,S | 1.8 | 12.4 | – | – | P | | D | 1 |
| 15 | 53 | M | D,S | 2.0 | 12.4 | 12.4 | – | S | 4 | A | 11 |

Sites: D, skin; H, liver; M, marrow; N, nodes; 1, regional; 2, peripheral (not 1); 3, mediastinal; 4, intra-abdominal; O, bone; P, pulmonary; S, soft tissue.
Overall response: P, progression ($P^1$, pulmonary metastases responded; $P^2$, skin metastases responded); PR, partial response, ( ) indicates course number that response was noted; S, stable; A, alive; D, dead.

**Table II** Patient details, phase II (11.0 × $10^6$ U $m^{-2}$) study

| Pat. no. | Age | Sex | Metastatic sites | Body surface area ($m^2$) | No. of courses given | Overall response w. course no. | Duration of stable disease or response | Status | Survival (months) |
|---|---|---|---|---|---|---|---|---|---|
| 16 | 45 | F | H | 1.7 | 3 | S | 16 | A | 16 |
| 17 | 52 | M | $N_{1,2,3}$,P,S | 1.6 | 2 | P | | D | 6 |
| 18 | 42 | M | O,P,S,SI | 1.9 | 3 | PR(1) | 4 | A | 11 |
| 19 | 56 | M | A,$N_2$,P,SP,S | 1.9 | 1 | P | | D | 2 |
| 20 | 52 | F | D,O,P | 1.5 | 1 | P | | D | 2 |
| 21 | 36 | F | O | 1.5 | 3 | S | 10 | A | 10 |
| 22 | 44 | M | $N_3$ | 2.0 | 3 | P | | D | 7 |
| 23 | 63 | F | D,O | 1.7 | 3 | S | 5 | D | 8 |
| 24 | 22 | F | D,H,P | 1.6 | 1 | P | | D | 2 |
| 25 | 43 | M | $N_3$,O,P | 1.9 | 2 | P | | D | 4 |
| 26 | 63 | F | D,H,P | 1.7 | 1 | P | | D | 1 |
| 27 | 69 | F | D,$N_1$,O,P | 1.6 | 3 | P | | D | 8 |
| 28 | 30 | M | D,$N_4$,O,P | 1.5 | 2 | P | | D | 5 |
| 29 | 55 | M | D,P | 1.6 | 2 | P | | D | 6 |
| 30 | 40 | M | D,S | 1.9 | 3 | PR(3) | 3 | A | 9 |
| 31 | 58 | F | D,P | 1.8 | 3 | P | | D | 5 |

See notes to Table I.
A, adrenal; SI, small intestine; SP, spleen.
Patient nos. 3, 8, 18, 19, 22, 25, 28, 29 and 31 received chemotherapy on progression, with static disease in 8, 18, 28 and 29.

*Interleukin-2*

The rIL-2 used in the study was kindly supplied by the Cetus Corporation (Emeryville, California). Specific activity was 3 × $10^6$ Cetus unit (U) per mg of protein and reconstitution was with sterile water followed by infusion in 5% detrose over 1 h.

The first dose of rIL-2 was infused by a syringe pump via a catheter positioned in the splenic artery. A femoral Seldinger approach using the Simmon's no. 1 cerebral catheter (William Cook, Denmark) for the catheterisation was employed. Contrast injection confirmed satisfactory position both before and after the rIL-2 infusion. After the 1-h infusion the catheter was removed under screen control. Further doses of rIL-2 were given intravenously via a peripheral vein over 1 h (controlled by a volumetric pump). The first intravenous dose was given 4 h after the intrasplenic dose and further i.v. doses at 48, 96 and 144 h, i.e. on alternate days. A complete treatment course therefore involved five rIL-2 administrations given over a 6-day period. Treatment was repeated to a maximum of three courses at 21-day intervals from the start of the rIL-2.

In the phase I study, 35 courses were given in 15 patients. Doses were escalated according to a modified Fibonacci scheme from 1 × $10^6$ to 16.4 × $10^6$ U $m^{-2}$. Doses were escalated after two patients had been entered at each dose level and also 'within' patients who had successfully completed treatment at the lower dose. Escalation continued providing no grade III or IV toxicity occurred as assessed by standard World Health Organization criteria. The rIL-2 was discontinued if grade III or IV toxicity occurred during a course. Any patient with grade IV toxicity did not receive further rIL-2 but patients with grade III toxicity continued on the treatment programme providing that the toxicity completely resolved or returned to grade I levels.

*Supportive care*

Patients received paracetamol every 6 h when required for pyrexia. No other routine supportive medication was used. Moderate to severe hypotension was treated conservatively or with an infusion of 500 $cm^3$ of normal saline over 30–40 min. On four occasions only hydrocortisone 100 mg i.v. as one dose was also given in three patients with marked hypotension. No pressor agents were used. No specialised monitoring was undertaken and patients were nursed on a general medical oncology ward. Regular records of the patients' general status, blood pressure, pulse and temperature were taken every 15 min (× 8) after rIL-2, then every 30 min (× 4), hourly (× 4), and thereafter 4 hourly, while on treatment.

Following relapse or progression chemotherapy with DTIC 250 mg $m^{-2}$ daily for 5 days and melphalan 15 mg $m^{-2}$ i.v. bolus day 3 was considered with other appropriate therapy as indicated.

*Response, toxicity evaluation and follow-up*

Standard WHO criteria were used to define objective response and toxicity (Miller *et al.*, 1981). The worst toxicity grade experienced during an rIL-2 course was recorded. In addition the time of onset and duration of side-effects were recorded. Full blood counts and routine biochemistry were performed at the time of rIL-2 administration and weekly ($\times 2$) between courses. The patients' performance scores (Miller *et al.*, 1981) were also assessed, immediately before rIL-2 and a month after the last course.

A complete response (CR) was to be recorded if all evidence of melanoma disappeared for at least 4 weeks. Partial response (PR) was defined as a decrease by at least 50% in the sum of the product of the longest perpendicular diameters of measured lesions for at least 4 weeks. Stable disease (SD) was recorded in the presence of tumour which did not qualify for partial response or disease progression. Progressive disease (PD) was defined as a 25% or greater increase in the sum of the product of the perpendicular diameters of measurable disease or the appearance of new lesions. Patients were evaluated for response 4 weeks after completion of therapy. In the presence of progressive disease rIL-2 treatment was discontinued. Subsequent evaluation occurred at 4–6-weekly intervals for 6 months and 2–3-monthly therafter.

### Results

*Phase I study*

In the escalating dose, phase I study 15 patients received a total of 35 (of a possible 45) rIL-2 courses (see Table I). Eight patients received all three courses. The median single dose was $7.0 \times 10^6$ U m$^{-2}$ giving a median dose per course of $35 \times 10^6$ U m$^{-2}$ with a range of 5–82.5. The median cumulative dose was $106 \times 10^6$ U m$^{-2}$ (range 5–191). No grade IV, life threatening toxicity occurred (see Table III). There were no dosage reductions or delays due to toxicity but 11 individual doses out of a possible 175 were omitted due to toxicity. One patient with hepatic and marrow metastases had grade 3 anaemia, thrombocytopenia and elevation of bilirubin. Mild transient elevations of bilirubin up to three times normal occurred on five other courses. Transient elevations of aspartate aminotransferase (AST) were more frequent, occurring on 14 courses with elevations of 1.26–4 times the upper limit of normal. No oliguria or marked renal impairment occurred. Gastrointestinal toxicity was only of moderate severity when experienced and responded to routine care but regular antiemetics were needed on two courses. The median onset of gastrointestinal toxicity was 2 h (range 1–6 h) after rIL-2 with a median duration of 3 h (range 1–20 h). Fever, median 39.2°C, range 37.6–40.6°C occurred on all courses except two and most patients felt chilled. Patients were symptomatic from the fever for 1–4 h (median 3 h) following rIL-2. No peripheral neurotoxicity was noted but some patients felt more than usually lethargic up to 3 h after treatment. Two patients gained weight (> 10% to < 15% of the pretreatment body weight). In one patient this was associated with pre-existing ascites.

There were three episodes of a dry, itchy desquamating rash lasting 2–4 days and three episodes of arthralgia and myalgia lasting up to 12 h. There was marked eosinophilia (> 20% of the total WBC) on 12 courses, but this was without apparent relation to rashes, arthralgia or myalgia. An autoantibody screen was routinely performed and, on four occasions only, antibodies to rheumatoid factor and cardiolipin were newly detected. No autoantibodies to thyroid, stomach, smooth muscle, etc., were detected. One patient shortly after splenic artery catheterisation developed symptoms and signs of a peripheral vessel embolism in the foot but there was rapid recovery following conservative management.

The most clinically relevant toxicity concerned dyspnoea and hypotension. There were two episodes of dyspnoea at rest requiring bronchodilator therapy and nine episodes of severe hypotension. The median onset of these side-effects was 2 h (range 1–8 h) after rIL-2, lasting for only 30 min to 2 h. The duration of any degree of dyspnoea or hypotension was longer: median 4 h (range 1–11 h). Five of the nine episodes of hypotension occurred with the 26 courses in which the rIL-2 dose was lower than $12.4 \times 10^6$ U m$^{-2}$. The remaining four episodes of severe hypotension and the two episodes of severe dyspnoea occurred during the nine courses when the rIL-2 dosage was $12.4 \times 10^6$ U m$^{-2}$ or higher.

*Phase II study*

On the basis of the previous dose ranging study, $11 \times 10^6$ U m$^{-2}$ was chosen as the maximum tolerable dose and used in the phase II evaluation. Thirty-six courses of a possible 48 were given with 19 individual doses omitted because of toxicity. The median cumulative dose was $140 \times 10^6$ U m$^{-2}$ (range 11–165). One patient with massive intra-abdominal disease including bilateral adrenal metastases developed temporary respiratory arrest following removal of the splenic catheter. No other grade IV toxicity occurred. The other main toxicities in the phase II study are shown in Table III. On four occasions anaemia (6.5–9.4 g%) occurred and on one occasion transfusion was required. Again transient elevations in AST and creatinine occurred on 13 courses with increases from 1.26 to less than 3-fold the upper limit of normal.

Although vomiting occurred on 17 courses it was transient and only two courses required regular antiemetics. The median onset of gastrointestinal toxicity was 2 h (range 1–5 h) with a median duration of 3 h (range 1–13 h). Diarrhoea and central nervous system side-effects were uncommon and only mild. Again, fever occurred on the majority of courses and on 18 courses was in excess of 40°C but the median maximum temperature was 39.2°C (range 37.4–40.3). The accompanying chills were only temporary, lasting for 0.5–3 h (median 2 h) after giving the rIL-2. With the first course of rIL-2 one patient had transient 'splenic' pain following the catheterisation. A desquamating rash occurred after three courses, lasting up to 48 h. On 22 courses there was some arthralgia and/or myalgia with a median onset of 3 h after the rIL-2 (range 1–8 h) lasting for a median of 3 h (range 1–24 h). Autoantibodies were not detected. Eosinophilia greter than 20% of the total white count occurred on 21 courses.

There were five episodes of severe hypotension other than the patient who had a transient respiratory arrest. This patient accounted for the sole episode of grade 4, pulmonary

**Table III** Toxicity

| | *Number of courses with WHO grades* | | | | | | | |
|---|---|---|---|---|---|---|---|---|
| | *Phase I (35)* | | | | *Phase II (36)* | | | |
| *Grade* | *0* | *1* | *2* | *3* | *0* | *1* | *2* | *3* |
| Haemoglobin | 12 | 15 | 6 | 2 | 29 | 3 | 3 | 1 |
| Leucocytes | 33 | 1 | 1 | – | 34 | 1 | 1 | – |
| Platelets | 33 | 1 | – | 1 | 35 | 1 | – | – |
| Bilirubin | 29 | 3 | 2 | 1 | 32 | 4 | – | – |
| AST | 21 | 9 | 5 | – | 26 | 7 | 3 | – |
| Creatinine | 33 | 2 | – | – | 33 | 2 | 1 | – |
| Nausea/vomiting | 12 | 5 | 16 | 2 | 4 | 15 | 15 | 2 |
| Diarrhoea | 30 | 4 | 1 | – | 31 | 4 | 1 | – |
| Fever | 2 | 4 | 24 | 5 | 2 | 8 | 8 | 18 |
| CNS | 25 | 7 | 3 | – | 31 | 5 | – | – |
| Pulmonary | 31 | – | 2 | 2 | 31 | 2 | 2 | 1 (grade 4) |
| Hypotension[a] | 16 | 3 | 7 | 9 | 18 | 8 | 4 | 6 (with one grade 4) |

[a]Hypotension grades: 1, > 20 mmHg systolic change or light headedness; 2, > 30 mmHg systolic change or orthostatic symptoms with pulse increase > 15 with upright posture; 3, > 40 mmHg systolic change or require fluid therapy.

toxicity and hypotension. Three other patients had an increase in blood pressure of between 10 and 20 mmHg. These side-effects were transient, again with a median onset of 3 h following rIL-2 (range 2–7 h) and a median duration of 5 h (range 2–13 h), for any degree of hypotension or dyspnoea.

*Response*

Four patients had a partial response but in only one of these did response occur in all metastatic sites. In the other three patients, partial response in one or more metastatic sites was associated with stable disease in other sites. The course number and rIL2 dose associated with the response were inconsistent. Durations of static disease and response are also given in Tables I and II. Another 19 patients had progressive disease and the remaining patients stable disease. Sites of response included skin (two patients), soft tissue (two patients), peripheral lymph nodes (one patient), mediastinum (one patient), parenchymal lung (one patient) and one patient each in liver and small bowel metastases (see Tables I and II). Nine patients received chemotherapy on progression (see Table II). There was no clear difference between the lymphocyte count during rIL-2 for patients whose disease progressed and those patients without progression (Figure 1). Median values and ranges of the lymphocyte count are displayed in Table IV. In some patients the performance score improved despite the advanced stage of disease in the patient population (Table V). The median survival of the whole group of 31 patients was 8 months with a range of 1–16 months. Six patients remain alive with stable disease.

**Figure 1** Lymphocyte count during IL-2 treatment. Continuous line, non-progression; dotted line, progression.

**Table IV** Lymphocyte count (cells $\times 10^6\,l^{-1}$)

| Non-progressors | | | Progressors | | |
|---|---|---|---|---|---|
| *Day* | *Median* | *Range* | *Day* | *Median* | *Range* |
| 0 | 1776 | 742–3150 | 0 | 1840 | 490–7769 |
| 2 | 819 | 165–5394 | 2 | 833 | 77–4950 |
| 7 | 1322 | 510–5804 | 7 | 1989 | 605–3096 |
| 14 | 2505 | 364–4958 | 14 | 4127 | 7000–5880 |
| 21 | 2035 | 350–9333 | 21 | 2557 | 700–4218 |
| 23 | 1150 | 158– 805 | 23 | 1260 | 371–7130 |
| 28 | 2156 | 1067–6594 | 28 | 1677 | 689–3213 |
| 35 | 3862 | 1804–6375 | 35 | 4125 | 1007–9730 |
| 42 | 2332 | 1416–7788 | 42 | 2382 | 869–4234 |
| 44 | 3939 | 1860–6195 | 44 | 4224 | 2220–5538 |
| 49 | 2584 | 936–3850 | 49 | 2160 | 1292–3105 |
| 56 | 3914 | 456–6030 | 56 | 3183 | 1474–4758 |
| 63 | 2185 | 1660–8418 | 63 | 2222 | 1056–5382 |

**Table V** Change in performance status with rIL-2

| | Phase I | | Phase II | |
|---|---|---|---|---|
| | *Pre rIL-2* | *One month after last rIL-2* | *Pre rIL-2* | *One month after last rIL-2* |
| PS 0 | – | 2 | – | 2 |
| 1 | 6 | 3 | 8 | 3 |
| 2 | 8 | 3 | 6 | 5 |
| 3,4[a] | 1 | 7(5) | 2 | 6(4) |

[a]Includes patients dying, number in parentheses, within 1 month of last course rIL-2.

PS: 0, normal activity; 1, ambulatory, can do light work; 2, ambulatory, self caring, unable to do any work; 3, limited self care, confined to bed or chair; 4, completely disabled, no self care. See Miller *et al.* (1981).

### Discussion

Interleukin-2 was recognised as a T cell growth factor by Morgan *et al.* in 1976. It was also noted later to be a differentiation factor for cytotoxic T cells and was found to activate natural killer cells and LAK cells (Grimm *et al.*, 1982). Adoptive immunotherapy in patients with advanced cancer with rIL-2 was first explored by Rosenberg *et al.* (1987). The rIL-2 was given intravenously three times a day as based on previous work using murine tumours (Rosenberg *et al.*, 1985). The administration in patients of doses of 100,000 units $kg^{-1}$ every 8 h (median cumulative dose per patient was $1.8 \times 10^6$ U $kg^{-1}$ or approximately $8.4 \times 10^7$ U $m^{-2}$) led to a variety of severe side-effects (Rosenberg *et al.*, 1987). The life threatening toxicity appeared to be largely due to a reduction in vascular resistance probably due to increased capillary permeability. The hypovolaemia also resulted in hypotension which on occasions led to other problems, e.g. renal failure or myocardial infarction. However, these first studies, demonstrated tumour regression, particularly in patients with advanced renal cell carcinoma and malignant melanoma. When rIL-2 was given as a continuous 24 h infusion ($3 \times 10^6$ U $m^{-2}$) for 5 days, the side-effects were much less and responses were again noted in good performance stage patients with melanoma (West *et al.*, 1987). It could be argued that a continuous infusion results in a sub-optimal tumour response due to low peak levels of rIL-2.

In our study using an alternate day regimen of high dose rIL-2 the cumulative dose delivered per patient of $10.6 \times 10^7$ U $m^{-2}$ in the phase I and $14.0 \times 10^7$ U $m^{-2}$ in the phase II study was comparable to that in the Rosenberg series. These cumulative doses were delivered over a median of three courses whereas in the Rosenberg schedule the cumulative dose of $8.4 \times 10^7$ U $m^{-2}$ was given per patient, the median number of courses administered being one. The 4 h i.v. dose following the intrasplenic dose was not given on 13 occasions, out of a total of 30 doses omitted. There was no evidence that the intrasplenic dose was associated with less toxicity than intravenous doses. We were also able to demonstrate that natural killer and LAK cell activity was induced in these patients (Ghosh *et al.*, 1989). The induction of killer cell activity may have resulted partly from the intrasplenic approach and in experimental models rIL-2 has induced both lymphoid proliferation in the spleen and LAK cell activity (Ettinghausen *et al.*, 1985). We were unable to demonstrate any relationship between lymphocyte count (total white and also eosinophil counts, data not presented) and response or survival despite considerable changes in these cell counts with treatment. West *et al.* (1987) noted that tumour response was associated with a good performance status, a pre-treatment lymphocyte count above 1,400 cells $mm^{-3}$ and an rIL-2 induced lymphocytosis of at least 6,000 cells $mm^{-3}$. It was suggested that a threshold lymphocytosis of 6,000 cells $mm^{-3}$ was associated with response and if this count was not obtained alternative treatment should be considered. Our data did not support the association begtween lymphocytosis (or elevation of the total white count or eosinophilia) and response.

Although we were unable to obtain any complete responses in our patients, the majority of whom had three metastatic sites involved, it was encouraging that response was seen in visceral sites. Some patients remain alive with stable disease who before rIL-2 therapy had progressing melanoma. Certainly, the treatment regimen was not associated with a high frequency of major side-effects and was generally well tolerated despite a patient population with very advanced disease and limited performance status. Furthermore, side-effects were few and transient and hospitalisation was only required for the rIL-2 therapy except in two patients.

Further experimental studies with rIL-2 are indicated as it is now possible to avoid serious side-effects and treatment can be given on a general medical ward without intensive care facilities. LAK cell generations may also be unnecessary and different schedules of rIL-2 either alone or in combination with other materials, e.g. flavone acetic acid which is known to act synergistically with rIL-2 in murine systems, can be considered (Wiltrout *et al.*, 1988). Other techniques currently being examined include the expansion of the tumour infiltrating lymphocyte population with rIL-2 using lymphocytes isolated from the patient's own tumour. The use of other cytokines and biological response modifiers, e.g. interferons or tumour necrosis factor, in combination with rIL-2 may also improve the antitumour effect.

The authors acknowledge Cetus Corporation, Emeryville, California for the generous donation of recombinant interleukin-2 for this study. Dr H. Dazzi was in receipt of a Swiss Cancer League Fellowship

### References

BALCH, C.M., SOONG, S.-J., SHAW, H.M. & MILTON, G.W. (1985). Analysis of prognostic factors in 4000 patients with cutaneous melanoma. In *Cutaneous Melanoma: Clinical Management and Treatment Results Worldwide*, Balch, C.M., Milton, G.W., Shaw, H.M. & Soong, S.-J. (eds) p. 321. J.B. Lippincott: Philadelphia.

CORNBLEET, M.A., McELWAIN, T.J., KUMAR, P. & 6 others (1983). Treatment of advanced melanoma with high dose Melphalan and autologous bone marrow transplantation. *Br. J. Cancer*, **48**, 329.

ETTINGHAUSEN, S.E., LIPFORD III, E.H., MULE, J.J. & ROSENBERG, S.A. (1985). Systemic administration of recombinant interleukin 2 stimulates *in vivo* lymphoid cell proliferation in tissues. *J. Immunol.*, **135**, 1488.

GHOSH, A.K., DAZZI, H., THATCHER, N. & MOORE, M. (1989). Lack of correlation between peripheral blood lymphokine activated killer (LAK) cell function and clinical response in patients with advanced malignant melanoma receiving recombinant interleukin 2. *Int. J. Cancer*, **43**, 410.

GRIMM, E.A., MAZUMDER, A., ZHANG, H.Z. & ROSENBERG, S.A. (1982). Lymphokine activated killer cell phenomenon. Lysis of natural killer resistant fresh solid tumour cells by interleukin 2-activated autologous human peripheral blood lymphocytes. *J. Exp. Med.*, **155**, 1823.

MASTRANGELO, M.J., BAKER, A.R. & KATZ, H.R. (1985). Cutaneous melanoma. In *Cancer Principles and Practice of Oncology*, DeVita, V.T., Hellman, S. & Rosenberg, S.A. (eds) p. 1371. J.B. Lippincott: Philadelphia.

MILLER, A.B., HOOGSTRATEN, B., STAQUET, M. & WINKLER, A. (1981). Reporting results of cancer treatment. *Cancer*, **47**, 207.

MORGAN, D.A., RUSCETTI, F.W. & GALLO, R. (1976). Selective *in vitro* growth of T lymphocytes from normal human bone marrows. *Science*, **193**, 1007.

ROSENBERG, S.A., MULE, J.J., SPIESS, P.J., REICHART, C.M. & SCHWARTZ, S. (1985). Regression of established pulmonary metastases and subcutaneous tumour mediated by the systemic administration of high-dose recombinant interleukin-2. *J. Exp. Med.*, **161**, 1169.

ROSENBERG, S.A., LOTZE, M.T., MUUL, L.M. & 10 others (1987). A progress report on the treatment of 157 patients with advanced cancer using lymphokine activated killer cells and interleukin 2 or high dose interleukin-2 alone. *N. Engl. J. Med.*, **316**, 889.

THATCHER, N., ANDERSON, H., JAMES, R., DAVENPORT, P. & CRAIG, P. (1986). Treatment of metastatic melanoma by 24-hour DTIC infusions and hemibody irradiation. *Cancer*, **57**, 2103.

THATCHER, N., LIND, M., MORGENSTERN, G. & 4 others (1989). High dose, double alkylating agent chemotherapy with DTIC, Melphalan or Ifosfamide with marrow rescue for metastatic malignant melanoma. *Cancer*, **63**, 1296.

WEST, W.H., TAUER, K.W., YANNELLI, J.R. & 4 others (1987). Constant infusion recombinant interleukin 2 in adoptive immunotherapy of advanced cancer. *N. Engl. J. Med.*, **316**, 889.

WILTROUT, R.H., BOYD, M.R., BLACK, T.C. & 3 others (1988). Flavone-8-acetic acid augments systemic natural killer cell activity and synergizes with rIL-2 for treatment of murine renal cancer. *J. Immunol.*, **140**, 3261.